# Association between In Utero arsenic exposure, placental gene expression, and infant birth weight: a US birth cohort study

**DOI:** 10.1186/1476-069X-12-58

**Published:** 2013-07-16

**Authors:** Dennis Liang Fei, Devin C Koestler, Zhigang Li, Camilla Giambelli, Avencia Sanchez-Mejias, Julie A Gosse, Carmen J Marsit, Margaret R Karagas, David J Robbins

**Affiliations:** 1 Department of Surgery, Molecular Oncology Program, Miller School of Medicine, University of Miami, Miami, FL 33136, USA; 2 Department of Pharmacology and Toxicology, Program in Experimental and Molecular Medicine, Geisel School of Medicine at Dartmouth, Hanover, NH 03755, USA; 3 Department of Community and Family Medicine, Geisel School of Medicine at Dartmouth, Hanover, NH 03755, USA; 4 Department of Molecular and Biomedical Sciences, University of Maine, Orono, ME 04469, USA; 5 Department of Biochemistry and Molecular Biology, Miller School of Medicine, University of Miami, Miami, FL 33136, USA; 6 Department of Surgery, Molecular Oncology Program, Department of Biochemistry and Molecular Biology, Sylvester Comprehensive Cancer Center, Miller School of Medicine, University of Miami, Miami, FL 33136, USA; 7 Current address: National Institutes of Health, National Human Genome Research Institute, Bethesda, MD 20892, USA

**Keywords:** Arsenic, AQP9, ENPP2, Birth weight, Placenta, Biomarker

## Abstract

**Background:**

Epidemiologic studies and animal models suggest that in utero arsenic exposure affects fetal health, with a negative association between maternal arsenic ingestion and infant birth weight often observed. However, the molecular mechanisms for this association remain elusive. In the present study, we aimed to increase our understanding of the impact of low-dose arsenic exposure on fetal health by identifying possible arsenic-associated fetal tissue biomarkers in a cohort of pregnant women exposed to arsenic at low levels.

**Methods:**

Arsenic concentrations were determined from the urine samples of a cohort of 133 pregnant women from New Hampshire. Placental tissue samples collected from enrollees were homogenized and profiled for gene expression across a panel of candidate genes, including known arsenic regulated targets and genes involved in arsenic transport, metabolism, or disease susceptibility. Multivariable adjusted linear regression models were used to examine the relationship of candidate gene expression with arsenic exposure or with birth weight of the baby.

**Results:**

Placental expression of the arsenic transporter *AQP9* was positively associated with maternal urinary arsenic levels during pregnancy (coefficient estimate: 0.25; 95% confidence interval: 0.05 – 0.45). Placental expression of *AQP9* related to expression of the phospholipase *ENPP2* which was positively associated with infant birth weight (coefficient estimate: 0.28; 95% CI: 0.09 – 0.47). A structural equation model indicated that these genes may mediate arsenic’s effect on infant birth weight (coefficient estimate: -0.009; 95% confidence interval: -0.032 – -0.001; 10,000 replications for bootstrapping).

**Conclusions:**

We identified the expression of *AQP9* as a potential fetal biomarker for arsenic exposure. Further, we identified a positive association between the placental expression of phospholipase *ENPP2* and infant birth weight. These findings suggest a path by which arsenic may affect birth outcomes.

## Background

The environmental toxicant arsenic poses a significant threat to adult human health [1,2]. Emerging evidence now suggests that arsenic exposure in utero also poses health risks to the developing fetus [1-3]. A number of epidemiological studies have found significant associations between prenatal arsenic exposure and adverse infant outcomes, such as infant mortality, low birth weight, and birth defects [4-8]. These health problems were most evident in individuals exposed to high-level arsenic [2]. Conclusions from these epidemiological studies are further supported by results from animal models [9,10]. Moreover, an inverse association between arsenic exposure and birth weight was found in individuals with lower exposure levels [11,12]. Despite the strong association between arsenic exposure and a range of child health concerns, the mechanisms by which arsenic elicits these effects remain elusive [3,13-16].

We recently reported that food sources, such as rice, also can contribute in exposing pregnant women to arsenic [17]. This finding, coupled with elevated arsenic levels detected in infant food [18-20], has raised serious public health and scientific concerns regarding the potential for relatively common fetal/early childhood exposure to arsenic. Validated human biomarkers will facilitate risk assessment for low-level arsenic exposure during fetal development [21,22]. In this study, we sought to develop such biomarkers, identifying relevant genes associated with low-dose arsenic exposure in an area of United States (New Hampshire) where a cohort of pregnant women used private wells with arsenic both above and below the current drinking water maximum contaminant level (MCL) of 10 μg/L [2]. We also subsequently associated the expression of these biomarkers to infant birth weight to provide insight into the mechanisms underlying the adverse effect of in utero arsenic exposure on infant health.

## Methods

### Ethics statement

All research involving human participants has been approved by The Committee for the Protection of Human Subjects (CPHS) - the Institutional Review Board at Dartmouth College (CPHS#20844). An informational brochure was provided to women around the time of their first prenatal visit. They were invited to participate in the study when their glucose challenge test was being requested (at around 24 to 28 weeks gestation). Written informed consent was given. All potential participants who declined to participate were not disadvantaged in any way by not participating in the study.

### Study cohort and arsenic measurement

The current study consisted of 133 pregnant women from New Hampshire, and was part of the ongoing New Hampshire Birth Cohort Study evaluating the impact of environmental factors on pregnancy and child health [17]. Demographic and lifestyle information was collected during prenatal visits. Spot urine samples were collected at approximately 24–28 weeks of gestation. Details of sample collection and arsenic measurement were described previously [17]. Briefly, urine samples were analyzed for arsenic species (As^III^, As^V^, DMA^V^, MMA^V^, and arsenobetaine) using a high-performance liquid chromatography (HPLC) ICP-MS system at the University of Arizona. The detection limit ranged from 0.1 μg/L to 0.15 μg/L for each individual arsenic species. Total urinary arsenic (U-As) was calculated by summing the concentrations of As^III^, As^V^, DMA^V^ and MMA^V^. Infant clinical characteristics, including birth weight, were recorded from the newborn’s medical record.

### Placenta biopsy preparation and processing

Placenta biopsies were obtained from study participants at the time of delivery by obstetrical staff. The placenta was placed on a sterile cutting surface with the umbilical cord exposed. To minimize heterogeneity by collection site, biopsies were taken at the base of the umbilical cord insertion, measuring roughly 1 cm deep and 1 – 2 cm across, and were placed immediately in RNAlater (Life Technologies). Care was taken to avoid maternal decidua as well as fibrous connective tissue and calcifications during sampling of the placenta parenchyma. Samples were refrigerated within 2 hours of collection and placed in a −80 degree freezer for long-term storage.

### RNA extraction and gene profiling using Nanostring

RNA extraction and subsequent gene profiling were performed using three similarly sized batches (44 or 45 samples per batch). A representative piece of each placenta sample (~ 200 mg wet weight) was homogenized in Tri Reagent (Molecular Research Center) using an electronic homogenizer. RNA extraction was performed following the manufacturer’s directions and further purified using RNeasy columns (Qiagen). RNA quality was assessed using an Agilent Bioanalyzer. 100 ng RNA from each sample was subject to gene expression analysis using the Nanostring system (Nanostring Technologies) at the Oncogenomics Core Facility of the University of Miami [23]. The Nanostring codeset was custom designed for 9 arsenic-related genes (*AKR1C3*, *ENPP2*, *HMOX1*, *LEP*, *NFE2L2*, *TYMS*, *AQP9*, *AS3MT*, and *SLC39A2*. Details in Table 1) and 5 house-keeping genes (*ATCB*, *GAPDH*, *HPRT1*, *RPL19*, and *RPLP0*). Raw data for the expression of each gene was compiled and normalized to the spike-in positive and negative controls using the nSolver software (Nanostring Technologies). The expression of arsenic-related genes was further normalized to the geometric mean of the expression of 5 house-keeping genes and presented as normalized counts per gene per sample.

**Table 1:** Candidate genes that were examined for their associations with in utero arsenic exposure

**Gene Symbol**	**RefSeq**	**Gene Name**	**Regulation by arsenic (biological system) [Reference]**
AKR1C3	NM_003739	aldo-keto reductase family 1, member C3	Up-regulation (cell line) [24]
ENPP2	NM_001040092	autotaxin	Up-regulation (cell line) [25]
HMOX1	NM_002133	heme oxygenase 1	Up-regulation (cell line) [26]
LEP	NM_000230	leptin	Up-regulation (placenta) [14]
NFE2L2	NM_001145413	nuclear factor (erythroid-derived 2)-like 2	Up-regulation (cell line) [27]
TYMS	NM_001071	thymidylate synthetase	Down-regulation (cell line, primary white blood cells) [28]
AQP9	NM_020980	aquaporin 9	Up-regulation (liver) [29]
AS3MT	NM_020682	arsenic (+3 oxidation state) methyltransferase	not available
SLC39A2	NM_014579	solute carrier family 39, member 2	not available

### Statistical analysis

The gene expression data from all 3 batches were first investigated for batch-to-batch variations using a principle components analysis. Batch effects were then adjusted by the COMBAT method [30]. Briefly, location (mean) and scale (variance) was adjusted using an empirical Bayes framework. Using the batch-adjusted data, a series of multivariable linear regression models were used to examine the association between U-As and gene expression for each of the candidate genes. Multivariable linear regression models modeled natural log-transformed gene expression as a function of log10-transformed U-As, and were adjusted for maternal age at delivery. Similar models were fit to investigate the association between *AQP9* expression and the expression of the other candidate genes as well as the association between gene expression and infant birth weight (kg). The following covariates were evaluated for inclusion in our models: maternal age, maternal smoking status (never, former, current), maternal education level, infant birth weight, infant gender and gestational age. Covariates controlled in our models were those that associated with the exposure and the outcome of interest using a series of linear regression models. A power analysis indicated adequate statistical power for detecting low-moderate correlations (absolute correlation (r) = 0.24; power = 80%) at a significance level of 0.05 and the study sample size of 133 subjects (Additional file 1: Figure S2). The aforementioned analyses were carried out using the R statistical program, version 2.13 (http://cran.r-project.org/). A structural equation model was applied to estimate the direct and indirect paths for U-As, birth weight, and the expression of *AQP9* and *ENPP2*[31]. This analysis is an extension of multivariable regression analysis that permits the simultaneous modeling of multiple dependent variables, allowing variables to be modeled both as dependent and independent with respect to other variables. We use this methodology to investigate the indirect relationship between arsenic exposure and infant birth weight. M-plus 6.12 software was used to estimate the coefficient estimates describing the associations along paths and their corresponding bias-corrected 95% bootstrap (10,000 replicates) confidence intervals.

## Results

The study group consists of 133 pregnant women enrolled in the New Hampshire pregnancy cohort (see Table 2 for demographic information). The mean infant birth weight was 3.4 kg (standard deviation (SD): 0.4 kg), similar to the national average [32]. The median arsenic concentration in household tap water was 0.36 μg/L (interquartile range (IQR) 0.02 – 3.55) with 84% of the participants having drinking water containing arsenic levels less than the current drinking water MCL of 10 μg/L. Total urinary arsenic concentration (U-As), incorporating both inorganic and organic metabolites (but not arsenobetaine), was also measured among the participants. The median concentration of U-As was 4.4 μg/L (IQR 1.8 – 11.9), which was consistent with an exposure range previously observed for a nationally representative US sample [33].

**Table 2:** Demographic information of the study group

**Characteristic**	**Value**^**a**^
Number of mother-child pairs	133
Mean gestational age (wks)	39.5 (1.6)
Mean maternal age at delivery (yrs)	31.1 (4.6)
Mean parity	1.1 (1.1)
Mean pre-pregnancy BMI (kg/m2)	24.9 (4.7)
Median tap water arsenic (/g/L)	0.36 {0.02 - 3.55}
Median U-As (/g/L)	4.4 {1.8 - 11.9}
School Level	
Less than 11th grade	3 [2.6]
High school graduate or equivalent	10 [7.5]
Junior college graduate or some college	28 [21.1]
or technical school	
College graduate	55 [41.4]
Post-graduate schooling	18 [13.5]
Unknown	19 [14.3]
Smoking status	
Never	97 [72.9]
Former	13 [9.8]
Current	5 [3.8]
Unknown	18 [13.5]
Mean infant birth weight (kg)	3.4 (0.4)
Infant gender	
Male	65 [48.9]
Female	68 [51.1]
Infant race	
White	128 [96.2]
Unknown	5 [3.8]

^a^Values are presented as mean (SD), number [%], or median {interquartile range}.

We used fetal portions of the placenta as a source of tissue for examining gene expression because of its fetal origin, its critical role in fetal development and control of the intrauterine environment, and easy accessibility after parturition. Maternal U-As was used as the parameter for fetal arsenic exposure, as arsenic has been shown to easily cross the placental barrier [34,35]. Thus, maternal U-As levels likely reflect the burden experienced by the fetus. To identify arsenic-associated biomarkers, we examined the expression of a set of previously reported arsenic-regulated genes (Table 1). Besides these putative arsenic target genes, the arsenic methyltransferase (*AS3MT*) and the zinc transporter (*SLC39A2*) were included because these genes were linked to arsenic-related health outcomes in previous studies [36-38].

**Figure 1:**
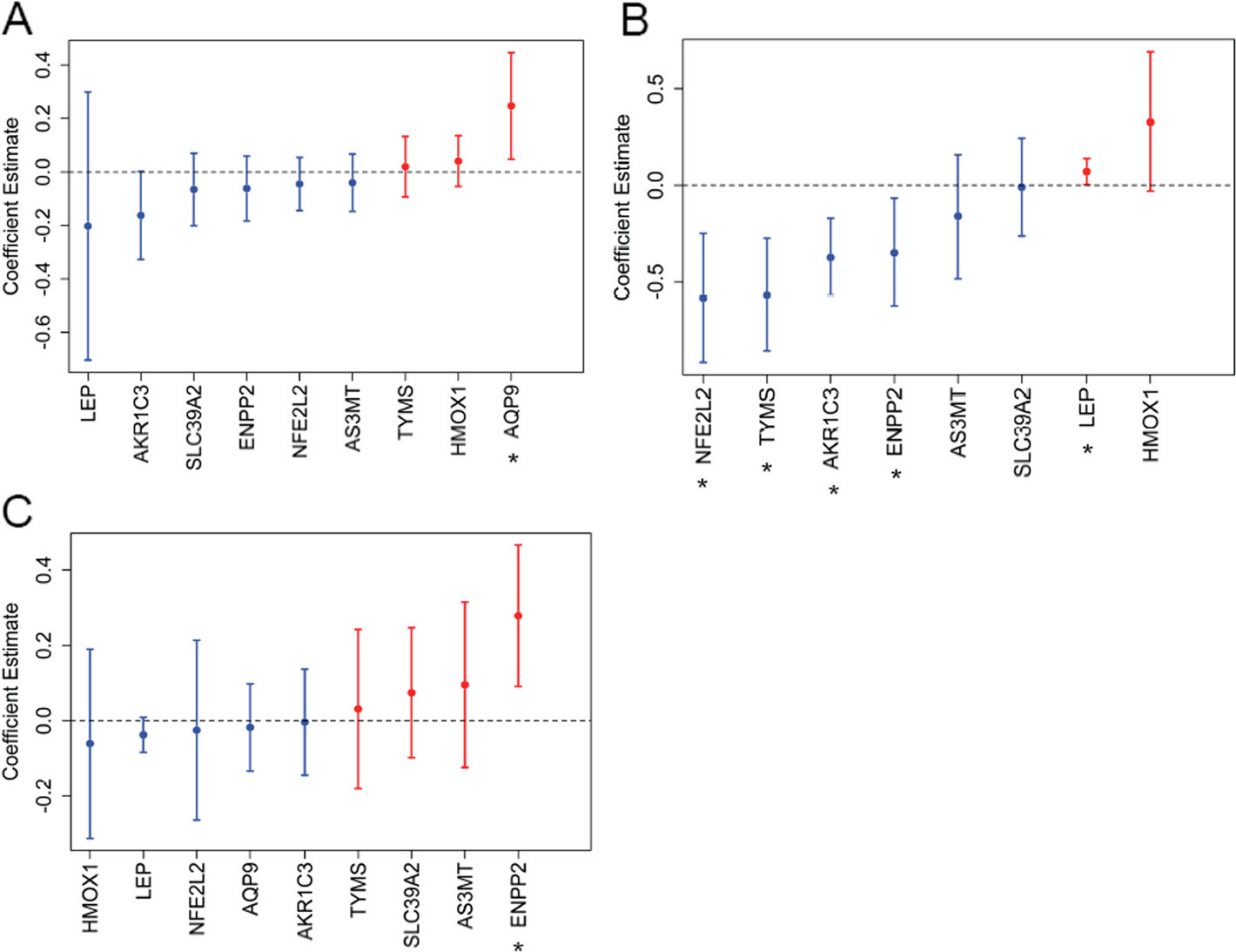
**Associations between gene expression, maternal U-As, and birth weight.** Multiple linear regression analyses for the association between (**A**) maternal U-As and placental gene expression, (**B**) *AQP9* expression and the expression of other genes, and (**C**) infant birth weight and gene expression. All analyses were adjusted for maternal age. The analysis for birth weight and gene expression was further adjusted for gestational age. Dots depict coefficient estimates and error bars represent 95% CIs. Positive coefficient estimates are marked in red and negative coefficient estimates are marked in blue. Significant associations are those with 95% CIs not crossing zero (dotted line) and are marked by asterisks.

After consolidating the candidate gene list, we first examined the relationship between the placental expression of these genes and in utero arsenic exposure. Multivariable linear regression models were used to investigate the association between the normalized expression value of each gene, quantitated using the Nanostring system [23], with maternal U-As. Among all the genes examined, the expression of *AQP9* was positively associated with arsenic exposure (coefficient estimate: 0.25; 95% CI: 0.05 – 0.45) (Figure 1A, Additional file 1: Figure S1 and Additional file 1: Table S1). Similar results were obtained when U-As was further adjusted for creatinine levels in samples where such data were available (Additional file 1: Table S2). We also evaluated whether other metal co-contaminants were related to both arsenic and *AQP9* and could be potential confounders in our analysis. While toenail Manganese (Mn) concentration showed the most significant association with arsenic levels from 88 participants in whom such data were available (Additional file 1: Table S3), the association between arsenic exposure and *AQP9* expression was not affected by adjustment of Mn concentrations (Additional file 1: Table S4).

As *AQP9* expression has been shown to enhance arsenic’s effect on cultured cells [39,40], presumably through increased arsenic uptake, we further determined the association between the expression of *AQP9* and the other members of our panel of putative arsenic biomarkers. Such analysis revealed significant associations between *AQP9* expression and five of the six arsenic-regulated genes in our panel (*AKR1C3*, *ENPP2*, *LEP*, *NFE2L2*, and *TYMS*), but not with the two genes not regulated by arsenic (*AS3MT* and *SLC39A2*) (Figure 1B and Additional file 1: Table S5). Further, as mentioned, in utero arsenic exposure has been linked to several adverse birth conditions such as low birth weight. To explore the mechanism by which arsenic exposure might influence birth weight, we examined the association between expression of our panel of candidate placental genes and infant birth weight. Multivariable linear regression analysis indicated that the expression of the phospholipase *ENPP2* was positively associated with increased infant birth weight (coefficient estimate: 0.28; 95% CI: 0.09 – 0.47) (Figure 1C and Additional file 1: Table S6). According to our analysis, a 10% increase in *ENPP2* expression was associated with 27 g increase in infant birth weight. Although not a primary aim of this study, we also observed an inverse association between U-As and infant birth weight in our cohort (coefficient estimate: -1.30, adjusted for infant gender, maternal age, and gestational age). The scale of arsenic effect on birth weight in our study group, -1.30 g per 1 μg/L increase in maternal U-As, was similar to those of previous studies [11,12].

**Figure 2:**
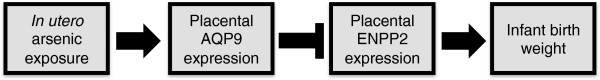
**Structural equation model for the effect of arsenic on birth weight.** The diagram illustrates the estimated path from arsenic exposure to birth weight. Variables are represented by rectangles. Each path is represented by a line with an arrow or vertical bar at one end. Arrow denotes positive association and vertical bar denotes negative association. The calculated coefficient estimate and 95% CI for each path are: in utero arsenic exposure (U-As) and placental *AQP9* expression (coefficient estimate: 0.25; 95% CI: 0.05 – 0.45); placental *AQP9* expression and *ENPP2* expression (coefficient estimate: -0.13; 95% CI: -0.22 – -0.02); placental *ENPP2* expression and infant birth weight (coefficient estimate: 0.28; 95% CI: 0.09 – 0.47). The indirect path from U-As to infant birth weight has a coefficient estimate of −0.009 and a 95% CI of −0.032 to −0.001 (n = 133 and 10000 replications for bootstrapping).

We further built a structural equation model to estimate how *AQP9* and *ENPP2* expression might contribute to arsenic-related affects on birth weight. Structural equation models have been successfully used for testing and estimating causal pathways and have been successfully employed for understanding the path from exposures to health outcomes [31,41-43]. For our study, this model incorporated the significant associations identified in this study to build a path from arsenic exposure to lower birth weight (Figure 2). According to this model, in utero arsenic exposure resulted in increased expression of *AQP9*, followed by decreased expression of *ENPP2*. Reduction in *ENPP2* levels ultimately associated with decreased infant birth weight. The indirect path from arsenic exposure to lower birth weight, through expression of *AQP9* and *ENPP2* expression, was statistically significant (coefficient estimate: -0.009; 95% CI: -0.032 – -0.001; 10,000 replications for bootstrapping), suggesting that exposure to arsenic in utero may be related to lower birth weight through this path.

## Discussion

From animal model experiments and epidemiologic studies from highly exposed populations, arsenic exposure has been associated with numerous adverse birth outcomes, such as low birth weight. However, the mechanisms by which arsenic induces these effects remain uncertain. Using placenta as the source of fetal tissue from a US cohort exposed to arsenic around the MCL, we identified the arsenic transporter AQP9 as a possible biomarker of arsenic exposure in fetal tissues. We subsequently identified a positive association between *ENPP2* expression and infant birth weight. We further developed a statistical path model whereby arsenic exposure related to lower infant birth weight through the modulation of *AQP9* and *ENPP2* expression. Thus, our model offers some insight into a possible mechanism underlying the inverse association between maternal arsenic exposure and infant birth weight.

AQP9 is an arsenic transporter [44,45]. Therefore, its increased expression has the potential to modulate the effect of arsenic on target cells. Consistent with this hypothesis, *AQP9* expression levels have been shown to modulate the effect of arsenic on cultured cells including those derived from human placenta [39,40]. Arsenic exposure, and resultant increased *AQP9* induced cytotoxicity of placental cells in theory could impact the function of the placenta, and thereby influence fetal growth and development. We speculated that increased placental *AQP9* expression also might enhance arsenic’s effect on fetal tissues. Indeed, *AQP9* expression was significantly associated with five of the six arsenic-regulated genes in our panel (Figure 1B and Additional file 1: Table S5). Interestingly, *AQP9* expression was negatively associated with the expression of *ENPP2*, *AKR1C3* and *NFE2L2*, in contrast to the increased expression previously reported in response to arsenic exposure in cultured mammalian cells (Table 1). U-As itself was inversely related to the expression of these same genes in our study cohort (Figure 1A, Additional file 1: Table S1 and Table S2). Thus, the inverse association between *AQP9* expression and *ENPP2*, *AKR1C3*, and *NFE2L2* expression could reflect a unique regulation by arsenic on these genes in human placenta. Besides arsenic, AQP9 transports solutes such as urea, glycerol, and monocarboxylates [46]. Thus, it remains possible that the observed associations between the expression of *AQP9* and some of the arsenic-related gene expression biomarkers may be indirect.

We hypothesize that arsenic exposure leads to lower birth weight by regulating the expression of *AQP9* and *ENPP2* based on the results of our structure equation model. Although direct evidence supporting this model is lacking, *AQP9* and *ENPP2* are expressed during human pregnancy under both physiologic and pathological conditions. For example, *AQP9* expression was elevated in placentas obtained from preeclamptic mothers [47]. *ENPP2* encodes a secreted phospholipase that catalyzes the conversion of lysophosphatidylcholine to lysophosphatidic acid (LPA) [48]. During pregnancy, the expression level of *ENPP2* in trophoblasts rises, contributing to the high circulating levels of ENPP2 and LPA in the serum of pregnant women [49,50]. LPA activates its cell surface receptors to regulate various processes relevant to reproduction, from angiogenesis and early embryonic development to embryo implantation and parturition, hence conceivably impacting birth weight [51]. Indeed, mice overexpressing lipid phosphate phosphatase 1 (LPP1), an enzyme that dephosphophorylates LPA, are born with lower birth weight [52]. Additionally, decreased levels of serum ENPP2 is associated with pregnancy-induced hypertension [53], which itself is associated with decreased infant birth weight [54]. While our findings suggest that placental expression of *AQP9* and *ENPP2* is related to in utero arsenic exposure and infant birth weight respectively, future research is necessary to validate these biomarkers in independent cohorts and to determine the precise molecular mechanisms of how arsenic exposure impacts birth weight through AQP9 and ENPP2.

## Conclusions

We have identified the expression of *AQP9* and *ENPP2* as novel potential fetal biomarkers, relating arsenic exposure to infant birth weight. As our results were based on a cohort of pregnant women exposed to arsenic levels above and below its MCL, these findings are relevant to pregnant women exposed to such levels of arsenic throughout the world.

## Abbreviations

CI: Confidence interval; IQR: Interquartile range; MCL: Maximum contaminant level; U-As: Total urinary arsenic concentration.

## Competing interests

The authors declare no competing interests.

## Authors’ contributions

DLF, JAG, MRK, and DJR designed research; DLF and CG performed research; DLF, DCK, AS, ZL, CJM, and MRK analyzed data; DLF, DCK, MRK, and DJR wrote the paper. All authors read and approved the final manuscript.

## Supplementary Material

Additional file 1: Table S1 Associations between maternal urinary arsenic concentrations during pregnancy (U-As) and the expression of placental genes. **Table S2.** Associations between maternal urinary arsenic concentrations during pregnancy (U-As) and the expression of placental genes after further adjustment of urinary creatinine levels in a subset of participants (n = 91). **Table S3.** The association between maternal toenail arsenic and other metals in a subset of participants (n = 88). **Table S4.** The association between maternal urinary arsenic concentrations during pregnancy (U-As) and *AQP9* expression with or without adjustment of toenail Mn levels in a subset of participants (n = 88). **Table S5.** Associations between the placental expression of *AQP9* and that of other genes. **Table S6.** Associations between placental gene expression and infant birth weight. **Figure S1.** Scattered plot view of the association between U-As and the placental expression of *AQP9*. **Figure S2.** Sample size power analysis. Click here for file
