# Supplementary material for: Association between In Utero arsenic exposure, placental gene expression, and infant birth weight: a US birth cohort study
Source: Environ Health. 2013 Jul 16;12:58. doi: 10.1186/1476-069X-12-58 (PMC3733767; doi:10.1186/1476-069X-12-58)
Supplement: Additional file 1: Table S1 — Associations between maternal urinary arsenic concentrations during pregnancy (U-As) and the expression of placental genes. Table S2. Associations between maternal urinary arsenic concentrations during pregnancy (U-As) and the expression of placental genes after further adjustment of urinary creatinine levels in a subset of participants (n = 91). Table S3. The association between maternal toenail arsenic and other metals in a subset of participants (n = 88). Table S4. The association between maternal urinary arsenic concentrations during pregnancy (U-As) and AQP9 expression with or without adjustment of toenail Mn levels in a subset of participants (n = 88). Table S5. Associations between the placental expression of AQP9 and that of other genes. Table S6. Associations between placental gene expression and infant birth weight. Figure S1. Scattered plot view of the association between U-As and the placental expression of AQP9. Figure S2. Sample size power analysis. [file 1476-069X-12-58-S1.pdf]

Supplemental Figure S1

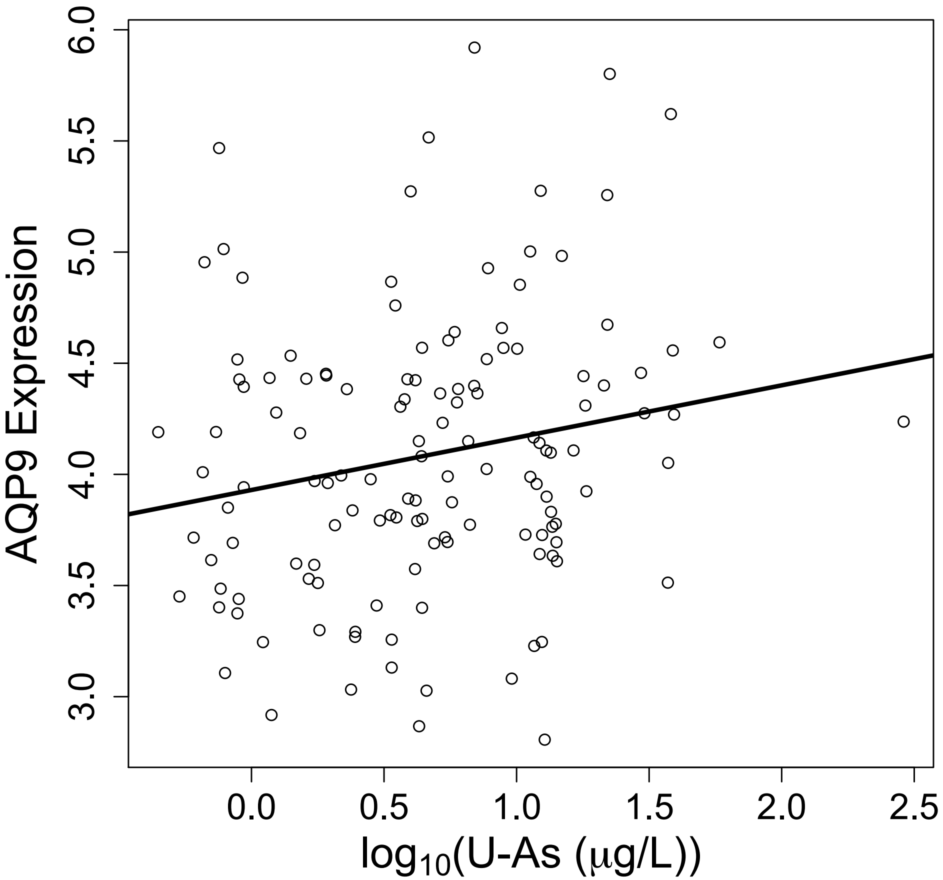

Scattered plot view of the association between U-As and the placental expression of *AQP9*.

Supplemental Figure S2

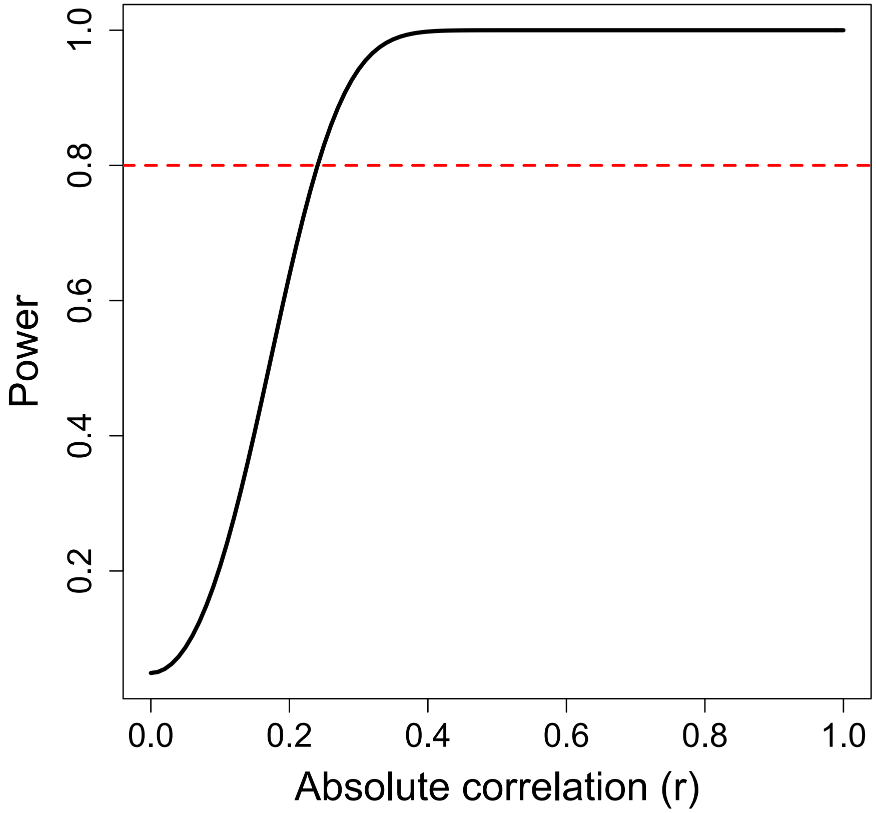

Sample size power analysis. Plot of power as a function of Pearson correlation (r), assuming the study sample size (n = 133) and a statistical significance threshold of 0.05. Red dotted line denotes 80% power.

Supplemental Table S1. Associations between maternal urinary arsenic concentrations during pregnancy (U-As) and the expression of placental genes.

Gene Expression	U-As[a]	
	Coefficient Estimate	95% CI
AKR1C3	-0.16	(-0.33, 0.0)
ENPP2	-0.06	(-0.18, 0.06)
HMOX1	0.04	(-0.05, 0.14)
LEP	-0.20	(-0.70, 0.30)
NFE2L2	-0.04	(-0.14 ,0.05)
TYMS	0.02	(-0.09, 0.13)
AS3MT	-0.04	(-0.15, 0.07)
AQP9	0.25	(0.05, 0.45)
SLC39A2	-0.07	(-0.20, 0.07)

[a]Adjusted for maternal age.

Supplemental Table S2. Associations between maternal urinary arsenic concentrations during pregnancy (U-As) and the expression of placental genes after further adjustment of urinary creatinine levels in a subset of participants (n =91).

Gene Expression	U-As[a]	
	Coefficient Estimate	95% CI
AKR1C3	-0.17	(-0.43, 0.09)
ENPP2	-0.15	(-0.33, 0.02)
HMOX1	0.08	(-0.06, 0.22)
LEP	-0.38	(-1.13, 0.38)
NFE2L2	-0.16	(-0.31, -0.02)
TYMS	0.00	(-0.17, 0.17)
AS3MT	-0.14	(-0.29, 0.02)
AQP9	0.43	(0.14, 0.71)
SLC39A2	-0.07	(-0.28, 0.14)

[a]Adjusted for maternal age and urinary creatinine levels.

Supplemental Table S3. The association between maternal toenail arsenic and other metals in a subset of participants (n = 88).

Metals	Pearson correlation	p-value
Al	0.20	0.0629
V	0.34	0.0013
Cr	0.01	0.9550
Mn	0.34	0.0012
Fe	0.19	0.0815
Ni	-0.20	0.0563
Cu	-0.15	0.1581
Zn	0.03	0.7842
Se	-0.17	0.1100
Mo	0.20	0.0594
Cd	0.16	0.1316
Sn	0.18	0.1023
Sb	0.13	0.2336
Hg	0.05	0.6746
Pb	0.09	0.4167
U	0.28	0.0081

Supplemental Table S4. The association between maternal urinary arsenic concentrations during pregnancy (U-As) and AQP9 expression with or without adjustment of maternal toenail Mn levels in a subset of participants (n = 88).

	Without Mn adjustment		With Mn adjustment	
Gene Expression	Coefficient Estimate	95% CI	Coefficient Estimate	95% CI
AQP9	0.18	(-0.08, 0.43)	0.18	(-0.07, 0.44)

Supplemental Table S5. Associations between the placental expression of *AQP9* and that of other genes.

Gene Expression	AQP9[a]	
	Coefficient Estimate	95% CI
AKR1C3	-0.25	(-0.39, -0.12)
ENPP2	-0.13	(-0.22, -0.02)
HMOX1	0.07	(-0.01, 0.15)
LEP	0.44	(0.02, 0.86)
NFE2L2	-0.14	(-0.22, -0.06)
TYMS	-0.18	(-0.27, -0.09)
AS3MT	-0.05	(-0.14, 0.05)
SLC39A2	0.00	(-0.12, 0.11)

[a]Adjusted for maternal age.

Supplemental Table S6. Associations between placental gene expression and infant birth weight.

Gene Expression	Birth Weight (kg)[a]	
	Coefficient Estimate	95% CI
AKR1C3	0.00	(-0.14, 0.14)
ENPP2	0.28	(0.09, 0.47)
HMOX1	-0.06	(-0.31, 0.19)
LEP	-0.04	(-0.08, 0.01)
NFE2L2	-0.02	(-0.26, 0.21)
TYMS	0.03	(-0.18, 0.24)
AS3MT	0.10	(-0.12, 0.32)
AQP9	-0.02	(-0.13, 0.10)
SLC39A2	0.07	(-0.10, 0.25)

[a]Adjusted for maternal age and gestational age.
